# Cracking the Code: Unveiling the Diversity of Carbapenem-Resistant *Klebsiella pneumoniae* Clones in the Arabian Peninsula through Genomic Surveillance

**DOI:** 10.3390/antibiotics12071081

**Published:** 2023-06-21

**Authors:** Amani H Al Fadhli, Shaimaa F. Mouftah, Wafaa Y. Jamal, Vincent O. Rotimi, Akela Ghazawi

**Affiliations:** 1 Laboratory Sciences, Department of Medical, Faculty of Allied Health Sciences, Health Sciences Center (HSC), Kuwait University, Jabriya 24923, Kuwait; 2 Department of Medical Microbiology and Immunology, College of Medicine and Health Sciences, United Arab Emirates University, Al Ain 15551, United Arab Emirates; 201590053@uaeu.ac.ae; 3 Department of Biomedical Sciences, University of Science and Technology, Zewail City of Science and Technology, Giza 12578, Egypt; 4 Department of Microbiology, College of Medicine, Kuwait University, Jabriya 24923, Kuwait; wafaa.jamal@ku.edu.kw; 5 Center for Infection Control and Patient Safety, College of Medicine University of Lagos, Idi-Araba 102215, Nigeria; bunmivr@yahoo.com

**Keywords:** genomic surveillance, CRKP, clone divergence, Arabian Peninsula

## Abstract

The rise of antimicrobial resistance is a global challenge that requires a coordinated effort to address. In this study, we examined the genetic similarity of carbapenem-resistant *Klebsiella pneumoniae* (CRKP) in countries belonging to the Gulf Cooperation Council (GCC) to gain a better understanding of how these bacteria are spreading and evolving in the region. We used in silico genomic tools to investigate the occurrence and prevalence of different types of carbapenemases and their relationship to specific sequence types (STs) of CRKP commonly found in the region. We analyzed 720 publicly available genomes of multi-drug resistant *K. pneumoniae* isolates collected from six GCC countries between 2011 and 2020. Our findings showed that ST-14 and ST-231 were the most common STs, and 51.7% of the isolates carried *bla*_OXA-48-like_ genes. Additionally, we identified rare carbapenemase genes in a small number of isolates. We observed a clonal outbreak of ST-231 in Oman, and four Saudi isolates were found to have colistin resistance genes. Our study offers a comprehensive overview of the genetic diversity and resistance mechanisms of CRKP isolates in the GCC region that could aid in developing targeted interventions to combat this pressing global issue.

## 1. Introduction

Antimicrobial resistance is a growing serious threat to human health and projected to reach an all-time high by 2050, resulting in millions of deaths and a massive economic burden [1,2]. Enterobacterales, including *Klebsiella pneumoniae*, are opportunistic pathogens responsible for many hospital-acquired infections [3]. Their broad spectrum of diseases and increasing resistance to antibiotics account for almost one-third of infections caused by Gram-negative bacteria. [3,4]. Resistance to carbapenems, the last resort class of antibiotics, is a major concern, especially in *K. pneumoniae* [5]. Studies suggest that the mortality rate associated with carbapenem-resistant *K. pneumoniae* (CRKP) may exceed 75% depending on factors such as age and disease profile [1,6]. The production of enzymes by genes that mediate different mechanisms of resistance in CRKP isolates effectively degrades carbapenems, rendering the bacteria non-susceptible [7]. *Klebsiella pneumoniae* carbapenemase (KPC), located on self-conjugative plasmids, is the most problematic class A carbapenemase due to its ability to spread widely [8]. The most common KPC enzyme alleles are KPC-2 and KPC-3 that are distributed globally [7,9]. Class B carbapenemases, metallo-β-lactamases (MBLs), are the second most significant enzymes in CRKP and can hydrolyze almost all β-lactam antibiotics, including carbapenems [10,11]. The most prevalent MBLs are IMP, VIM, NDM, GIM, and SIM which are located on genetic mobile elements that can transfer between bacteria. VIM and NDM are the most common globally, including in the Gulf [7,12]. NDM-1 is highly transferrable and can hydrolyze all β-lactams except aztreonam. It is primarily found in India [13]. VIM has more than 24 allelic variations in over 60 species [14]. Class D β-lactamases, such as the OXA-48-like enzyme, are commonly present in the Enterobacterales family and have significantly contributed to the rise of carbapenem resistance in the past decade [15]. The most significant reservoirs of these enzymes are India, the Middle East, and North African countries [16]. OXA-48 variants, including OXA-48, OXA-181, OXA-232, OXA-204, OXA-162, OXA-163, and OXA-244 have been identified [17].

CRKP is a major concern in the Gulf region, with specific clonal lineages identified, including the famous CC258 clone (ST258, ST11, ST340, ST437, and ST512) and other clones such as CG14/15, CG17/20, CG29, CG37, CG43, CG101, CG147, CG152, CG231, CG307, and CG490 [18]. However, the mechanisms of resistance and locally prevalent clones have only been explored through small-scale, local research. Studies such as [12,19,20,21,22,23,24,25,26,27,28,29,30] have contributed to our understanding of CRKP in the Gulf region. These studies have provided important information about the prevalence of specific clones and their resistance mechanisms in the region. However, larger-scale studies are needed to fully understand the impact of CRKP in the Gulf region and to develop effective prevention and treatment strategies.

Previous studies on the mechanisms of antimicrobial resistance (AMR) have focused on identifying only a few genes or mutations, while whole-genome sequencing (WGS) technology has improved the identification of various genetic bases of phenotypic variation, including point mutation, mobile genetic elements, and chromosomally encoded factors that contribute to the development of resistance, particularly to multiple antibiotics, leading to the emergence of MDR pathogens [31]. WGS data also allow identification of the evolutionary histories of homogeneous clusters using single nucleotide polymorphisms (SNPs) and the upscaling of multilocus sequence typing (MLST) perception using core genome MLST (cgMLST) analysis that provides better resolution and serves as the foundation for a universally curated nomenclature scheme accessible via various databases, enabling local and global epidemiological investigations [32,33].

To gain a better understanding of the genetic makeup of CRKP in the Gulf region, we employed cutting-edge techniques such as whole-genome sequencing and cgMLST analysis. Our investigation involved a comprehensive analysis of publicly available CRKP genomes that allowed us to identify the prevalence of various types of carbapenemases and their relationship with different sequence types (STs). By leveraging the power of in silico analysis, we were able to decipher the genetic relatedness of CRKP isolates in the Gulf Cooperation Council Countries region (GCCC) and gain insights into their evolution and spread. Our findings could pave the road to develop effective strategies to tackle the threat of CRKP and other multi-drug resistant pathogens in the GCCC and beyond.

## 2. Results

### 2.1. Prevalence and Distribution of Carbapenem-Resistant K. pneumoniae Sequence Types (STs)

As shown in Table 1, ST-14 represented 14.58% (105 out of 720) of the total isolates collected across all participant countries. The prevalence rates of ST-14 in the individual countries were UAE (*n* = 54, 7.5%), Saudi Arabia (*n* = 43, 6.0%), Qatar (*n* = 4, 0.6%), Oman (3, 0.4%), and Bahrain (*n* = 1, 0.1%). Of the 105 ST-14 isolates, 80% (84) carried at least one common carbapenemase gene. ST-231 was the second most common, accounting for 12.6% (91) of the total isolates. Amongst these 91 isolates, 74.7% (68) were found in Oman, 9.9% (9) in UAE, 8.8% (8) in Kuwait, and 6.6% (6) in Qatar (Table 1). Interestingly, ST-231 was absent in the Saudi Arabia collection. Moreover, various carbapenemase genes were detected among many isolates of this ST (73.6%; 67 out of 91) as shown in Table 1.

The subsequent frequently occurring ST was ST-2096, which accounted for 13.9% (100). Almost all these ST-2096 isolates (98%) were exclusively found in Saudi Arabia, specifically 98 out of 230 (42.6%) isolates from Saudi Arabia collection (Table 1). This ST was not detected in any other countries, except Qatar where only two (0.9%) isolates were identified. The carbapenemase genes of this lineage detected have only been found in Saudi Arabia where 87 (88.7%) of the isolates carried these resistance genes. The two isolates from Qatar did not contain any carbapenemase genes.

Out of the 720 ST identified, 76 (10.6%) belonged to the global epidemic clone ST-11. This clone was mainly identified in Oman (81%; *n* = 62), followed by UAE (7.8%; *n* = 6) and Qatar (6.5%; *n* = 5), with only three isolates detected in Saudi Arabia (3.9%; *n* = 3) (Table 1). The majority of these ST-11 isolates, 72 out of 76 (94.7%), produced carbapenemases as shown in Table 1.

Some of the STs such as ST-15, ST-101, ST-307 and ST-48 were detected in low proportions among the collections from each of the GCC states. ST-15 accounted for 25 (3.5%) of the total isolates and was found in five out of the six Gulf countries investigated (Oman, *n* = 10; Kuwait, *n* = 2; Saudi Arabia, *n* = 4; UAE, *n* = 7; Qatar, *n* = 2), with the majority, 21 out of 25 (84%), of these isolates carrying carbapenemase genes (Table 1). ST-101, on the other hand, was associated with only three out of the six countries studied (Saudi Arabia, *n* = 15; Oman, *n* = 8; Qatar, *n* = 2) (Table 1). ST-101 carbapenemase producers (88%; 22 out of 25) were found only in isolates from Saudi Arabia (*n* = 14) and Oman (*n* = 8). Similarly, ST-307 (*n* = 18) was observed in the same three countries (Saudi Arabia, *n* = 8; Qatar, *n* = 8; Oman, *n* = 2) (Table 1). ST-48 (*n*= 10) was identified in three countries (Saudi Arabia, *n* = 7; UAE, *n* = 1; Qatar, *n* = 2), while ST-45 was equally identified in only two countries (i.e., Saudi (*n* = 6) and Qatar (*n* = 6)) (Table 1).

### 2.2. Resistome Characterization of Carbapenem-Resistant K. pneumoniae Isolates

Carbapenem resistance genes were detected in 514 out of 720 (71.3%) of isolates (Table 2) and out of which 266 (51.7%) carried various alleles of *bla*_OXA_ genes alone and 159 (31%) carried *bla*_NDM-1_. A total of 11% (57/514) of isolates co-produced *bla*_NDM-1_ and multiple alleles of *bla*_OXA_. A small number of 12 (2%) co-produced *bla*_NDM-5_ and different alleles of *bla*_OXA_. Rare carbapenemase genes found in this study were *bla*_KPC-2_ (*n* = 10), *bla*_NDM-5_ (*n*= 5), *bla*_KPC-2_ in combination with *bla*_OXA232_ (*n* = 1), *bla*_KPC-3_ (*n* = 1), *bla*_NDM-7_ (*n* = 1), and *bla*_VIM-29_ (*n* = 2), with the latter found exclusively in Saudi Arabia.

### 2.3. Mobile Colistin Resistance Elements (mcr)

Only four isolates from Saudi Arabia, belonging to ST-2096 (one isolate), ST-14 (one isolate), and ST-3513 (two isolates), were found to contain the mcr and/or mcr-8 genes that conferred colistin resistance. The ST-14 isolate also carried a *bla*_NDM-1_ carbapenemase gene.

### 2.4. Clonal Clustering and Relatedness of the CRKP Isolates

**Figure 1:**
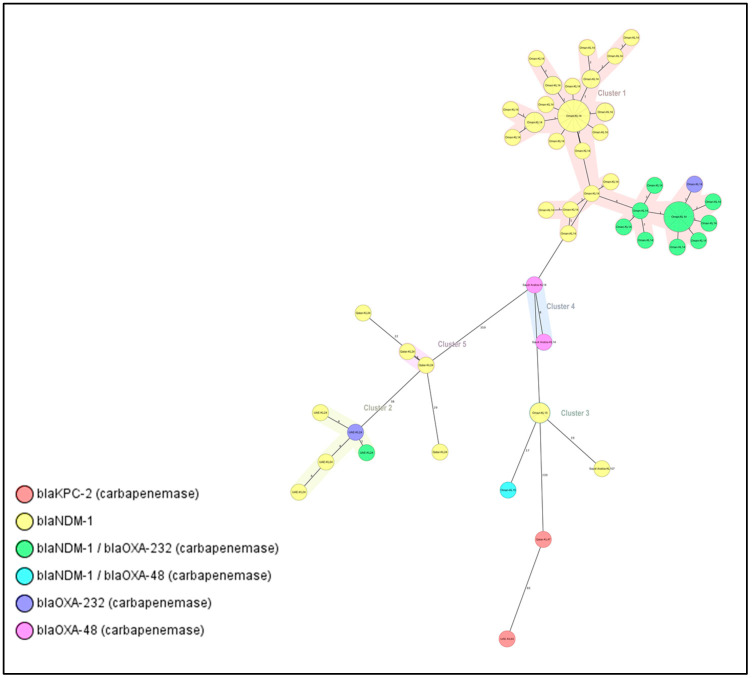
Minimum spanning tree (MST) of the 72 carbapenemase producers, ST-11 isolates. Distance based on the number of differences of the 2358 alleles in *K. pneumoniae* sensu-lato cgMLST. MST cluster distance threshold set at 15. Nodes labelled by column: Country of isolation and capsular type. Nodes colored by column: Carbapenem resistance gene.

cgMLST analysis was performed on isolates of the most abundant STs carrying at least one carbapenemase gene. The results indicated a clonal spread of certain STs within the same country or across the Arabian Peninsula. For instance, 72 out of 76 ST-11 isolates were carbapenemase producers, and five clusters and six singletons were identified. All clusters were made up of isolates from the same country. Cluster 1 consisted of 54 isolates (from Oman with less than 10 allele differences and KL14 as the dominant capsular type) (Figure 1). The majority of these isolates (*n* = 21) carried the *bla*_NDM-1_ gene, and nine had both *bla*_NDM-1_ and *bla*_OXA-232_ genes and clustered with one isolate with *bla*_OXA-232_, indicating clonal spread. Cluster 2 includes isolates from UAE with different carbapenemase genes and the same capsular type KL24. Other capsular and O antigen types were also present.

### 2.5. Clusters and Singletons Associated with ST-14 and ST-147

**Figure 2:**
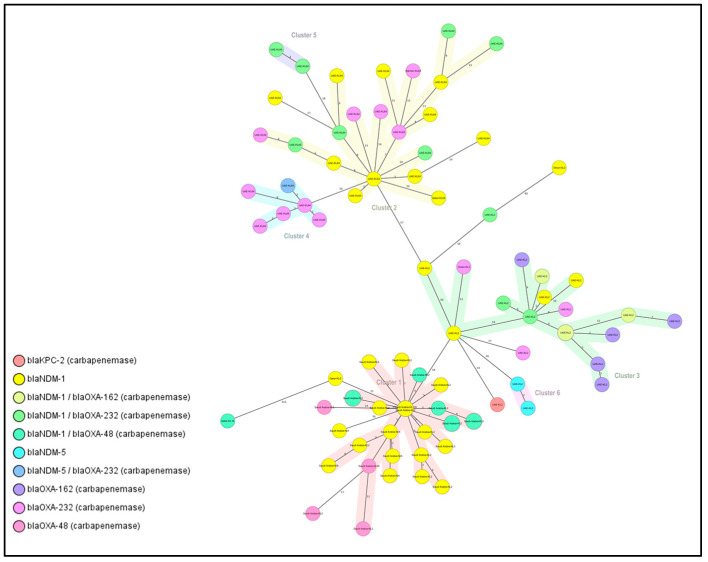
Minimum spanning tree (MST) of the 84 isolates (ST-14 carbapenemase producers). Distance based on the number of differences of the 2358 alleles in *K. pneumoniae* sensu-lato cgMLST. MST Cluster distance threshold set at 15. Nodes labelled by column: Country of isolation and capsular type. Nodes colored by column: Carbapenem resistance gene.

cgMLST analysis of 84 ST-14 carbapenemase producers revealed six clusters and eight singletons associated with ten different types of carbapenemase genes as shown in Figure 2. Most clusters contained isolates from the same country, with Cluster 1 being the largest (31.3%) and all from Saudi Arabia. Within this cluster, 10 isolates had *bla*_NDM-1_ and KL2; KL64 was the most common capsular type (Figure 2; Table 3). Cluster 2 contained isolates from UAE, Qatar, and Bahrain with different carbapenemase genes, while Cluster 3 had isolates from UAE and Oman. These findings suggest a clonal spread of this lineage with a high capability of acquiring different resistance genes and disseminating across different geographic locations.

cgMLST analysis of 58 ST-147 isolates found four clusters and 14 singletons, with eight different types of carbapenemase genes present. KL64 capsular type and O2a antigen were most common (Table 3). Cluster 1 and 2 had the most MST nodes, with isolates from different countries. The most frequent genes identified were *bla*_NDM-1_ in Cluster 1 and *bla*_NDM-5_ and/or *bla*_OXA-181_ in Cluster 2. Singletons from different locations were closely related with less than 45 allele differences, indicating a clonal expansion of this clone

### 2.6. Outbreaks Associated with ST-231 and ST-2096 CRKP

**Figure 3:**
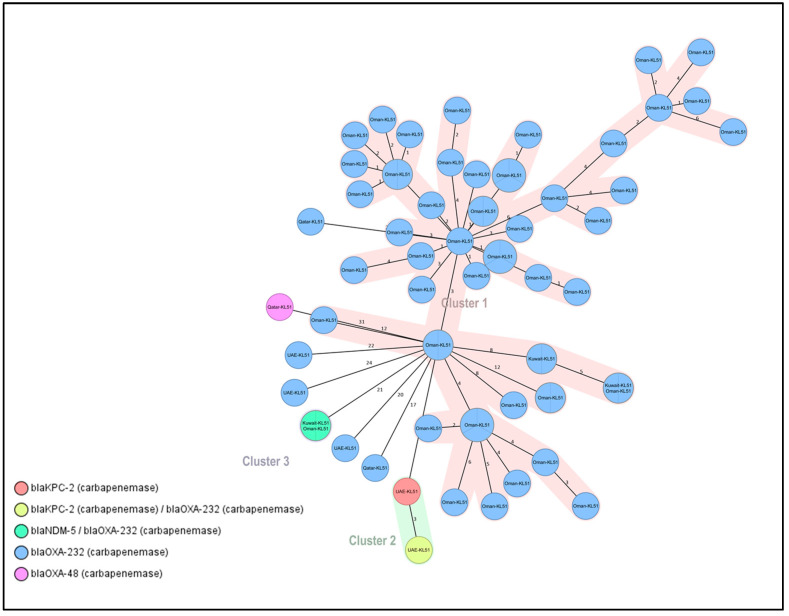
Minimum spanning tree (MST) of the 67 isolates (ST-231 carbapenemase producers). Distance based on the number of differences of the 2358 alleles in *K. pneumoniae* sensu-lato cgMLST. MST Cluster distance threshold set at 15. Nodes labelled by column: Country of isolation and capsular type. Nodes colored by column: Carbapenem resistance gene.

ST-231 isolates were found to be predominantly from Oman, with only a few from Kuwait, and all carried the *bla*_OXA-232_ gene. These isolates had the KL51 capsular type and O1 antigen (Table 3, Figure 3). ST-231 was found to be able to accommodate various carbapenem resistance genes as seen in Clusters 2 and 3, which contained isolates from different origins carrying different genes (Figure 3). Similarly, a clonal outbreak of ST-2096 CRKP was observed in Saudi Arabia in 2018, with most isolates having the KL64 capsular type and O1 antigen carrying either *bla*_OXA-48_ or *bla*_OXA-232_ genes. Cluster 1 contained the majority of isolates and had 67 MST nodes, with 19 carrying *bla*_OXA-48_ and 61 carrying *bla*_OXA-232_ (Table 3).

### 2.7. Clustering of Isolates among ST-15, ST-101, and ST-45 Lineages

In the ST-15 and CR-Kp-ST-101 lineages, carbapenemase producer isolates were grouped into clusters and singletons. Most isolates were from the same country except for Cluster 1. ST-15’s Cluster 1 included isolates from UAE and Kuwait with the *bla*_NDM-1_ gene, while ST-101’s Cluster 1 had isolates from Saudi Arabia and Oman with the *bla*_OXA-48_ gene. Different carbapenemase gene combinations were identified within each lineage. O1 was the predominant O antigen in ST-15 isolates, while KL17 capsular type and O1 antigen were common in ST-101 isolates. ST-45 was associated with *bla*_OXA-48_ and only two capsular types (KL43 and KL102) and one O antigen type (O2a) (Table 3).

## 3. Discussion

This study marks a cutting-edge initiative to investigate the genomic epidemiology of CRKP in the GCC countries. The findings revealed a dangerous clan that possesses both hyper-virulent and drug-resistant traits (specific STs and capsular types). Given the widespread distribution of these clones, they pose a significant public health risk [34]. Therefore, genetic epidemiology data from this study can provide insights into the evolution and complexity of these clones. Notably, the study identified that most of the isolates belonged to the ST14 clone, which has been previously recognized as a global threat [17,35].

The OXA-48-like carbapenemases are well known for causing outbreaks that affect specific sequence types, including ST14. Recently, Mouftah and colleagues (2021) reported the prevalence of this clone along with its associated clonal transmission and potential for horizontal gene transfer among isolates from 13 hospitals in the United Arab Emirates, Bahrain, and Saudi Arabia [36]. This clone has been detected in various regions globally, including Europe, the Mediterranean, China, North America, Oceania, and South Africa [17,35]. Several studies have also identified ST14 as one of the most common sequence types of NDM-1-producing *K. pneumoniae* (NPKP) [37,38,39]. It seems that our findings have been further substantiated as it appears that most of our isolates carried the NDM-1 and OXA-48 carbapenemase genes. Moreover, *K. pneumoniae* ST14 from the Arabian Peninsula also exhibited specific traits, such as the KL64 capsular locus and O1 antigens [36]. Alarmingly, an apparent outbreak of this clone in UAE with dominance of Hv capsular KL2 and KL64 has been identified based on cgMLST analysis [40,41]. In a related occurrence, an outbreak that initially took place in Saudi Arabia spread to Qatar with the dominance of KL2. Given that this clone has demonstrated an ability to carry multiple types of carbapenemase genes, it is clear that this Hv clone is becoming increasingly dominant and thus requires strict monitoring measures.

It is interesting to note that in our study, despite ST231 being the most common sequence type reported in previous cases of *bla*_OXA-232_-harboring *K. pneumoniae* [42], ST231 was in fact the second most prevalent sequence type. Additionally, we observed a higher occurrence of ST231 in Oman, which is not commonly seen in other countries in the region. Through our cgMLST analysis, we were able to identify an outbreak of this clone in Oman that had not been previously documented. Despite being located in different regions, this lineage has been strongly associated with locus type KL51 while carrying various carbapenemase genes as reported in other studies [43,44,45]. A recent study conducted in India also revealed a strong correlation between KL51 and ST231 in the phylogenetic tree of 307 isolates [46].

Our study revealed that a significant proportion of the *K. pneumoniae* isolates from Saudi Arabia were highly virulent (capsular serotype KL64) [40,41] and resistant, with the clonal complex 14 dominant sequence type being ST2096. These findings align with a previous outbreak in Saudi Arabia where an analysis of *K. pneumoniae* dissemination and transmission patterns identified a two-year outbreak of ST2096 beginning in December 2016 [47]. Additionally, we discovered that the epidemic clone ST11 was present in four out of six GCC countries, posing a significant threat to human health due to its carbapenem-resistant and highly transmissibility [48]. Recently, five CRKP isolates from the Oman outbreak were confirmed to belong to ST11 clones and were closely related to Chinese isolates from the Bigsdb (Bacterial Isolate Genome Sequence Database) [49]. Studies on ST diversification have yielded conflicting results, but most countries have prioritized monitoring the occurrences of ST11, ST15, and ST14 [50]. Notably, ST11 and ST14 were the most commonly reported STs in Asia, Europe, America, Africa, and Oceania, with ST11 being a common type in many studies [51,52,53]. Essentially, this means that the clone has the impressive power to gather diverse resistance genes as it spreads. In addition, scrutinizing the capsular locus divulged that all ST11 *K. pneumoniae* had unique K types including KL64. These discoveries align with prior research conducted in China where a thorough examination of 364 ST-11 isolates was carried out and published by Liu et al., 2022 [54].

The most common carbapenemases found in this collection were NDM and OXA-48-like enzymes, which are commonly found in CRE from the Arabian Peninsula, according to studies conducted by Jamal et al., 2016; Sonnevend et al., 2015; and Memish et al., 2015 [12,23,26]. The Arabian Peninsula and the Indian subcontinent have close socioeconomic interactions, resulting in similar resistance patterns. ST11 and ST258 strains, which are typically found in China and the US, are prevalent in both regions with a low incidence of KPC enzymes. However, KPC was detected in this collection, specifically in the ST15 clones rather than ST11 clones, as reported by Boyd et al., 2020 [55]. To our surprise, our ST101 isolate possessed the carbapenemase gene *bla*_VIM-29_, and this finding is significant as this clone has never been documented as producing VIM-29 before.

Our study revealed an intriguing aspect: the detection of *bla*_KPC-3_ exclusively among the ST258 clones from Qatar isolates. While KPC was previously rare in the Arabian Peninsula, a small number of isolates associated with recent medical care abroad were found to carry the gene according to Abid et al., 2021 [56]. These findings have significant regional and global implications, given Qatar’s demographics and its status as a major international travel hub. Although KPC is prevalent overseas, its emergence in the region requires attention due to its potent hydrolytic action and potential for dissemination.

Our findings indicate that only isolates from Saudi Arabia contain mobile colistin resistance elements (*mcr*). A recent study on the prevalence and molecular epidemiology of colistin-resistant Gram-negative bacilli (GNB) in Saudi Arabia revealed that while colistin still works well against GNB isolates locally, high levels of colistin resistance have been detected among major GNB such as *K. pneumoniae* [57]. Local data suggest that religious gatherings play a crucial role in triggering the acquisition of colistin resistance, and underscoring the importance of screening for colistin resistance determinants to prevent the spread of colistin-resistant GNB. Due to the absence of the most effective broad-spectrum antimicrobial agents, it is anticipated that colistin resistance will become more widespread shortly. Furthermore, mutations in the *mgrB* gene and insertion sequence transpositions were the most common mechanisms of colistin resistance among *K. pneumoniae* in the Middle East as well as in other regions [27,57].

Our analysis using cgMLST analysis showed that the most prevalent capsular type among *K. pneumoniae* was KL2, belonging to the ST14 clonal lineage. This finding is worrisome as this clonal lineage is associated with severe infections such as septicemia, pyogenic liver abscess syndrome, and pneumonia [58,59]. It has been suggested that both virulence and drug resistance are important for the pathogenesis of *K. pneumoniae* infections [60,61]. In hospital and community environments, *K. pneumoniae* clonal lineages have varying abilities to acquire resistance and virulence genes [62]. Therefore, it is crucial to have genomic surveillance in close geographic areas to understand the local epidemiology of *K. pneumoniae* infections in the Arabian Peninsula. Our cgMLST analysis also revealed distinct differences in the dominant high-risk STs of *K. pneumoniae* circulating in the GCC countries, with ST-11 being more prevalent in Oman, ST-14 in Saudi Arabia and the UAE, ST-147 in Qatar, Oman, and the UAE, and ST231 in Oman and Kuwait. Our study reveals that ST231 strains of *Klebsiella* had the highest presence of capsule genes/loci with KL51 being the most prevalent type [63]. On the other hand, the ST101 isolates of *K. pneumoniae* had KL17 capsular type and O1 antigen, which is consistent with previous reports linking pandemic ST101 with variants of KL17 and O1v1 [64]. Notably, the O1 antigen has been strongly linked to the virulence of *K. pneumoniae* in causing pyogenic liver abscesses [65].

Our study revealed a wide distribution of high-risk STs (ST-11, ST-14, ST-147, and ST-15) across each country in the region, which is consistent with previous observations [62,66]. These STs are prevalent in Asian countries, particularly India and the Philippines [67], which have a high percentage of workers in the Gulf region, suggesting that there may be multiple origins for the circulating lineages. The emergence of these high-risk clones may involve a complex phenomenon, including international transfer of successful clones and local dissemination of genetically flexible clones. Our cgMLST analysis revealed an association between some clones (ST-11, ST-14, ST-147, and ST-15) and carbapenemase genes (NDM-1), resulting in regional and local accessory gene sharing. Furthermore, the sharing of accessory genes within a local gene pool is increasing. These findings suggest that high-risk lineages are co-circulating and may have followed divergent evolutionary paths.

## 4. Materials and Methods

Demographic data. The population of the GCCC is one of the highest growing populations in the world owing primarily to immigration. The GCCC region includes six countries: (https://worldpopulationreview.com/country-rankings/gulf-countries (accessed on 20 January 2022).

Selection of bacterial isolates and data collection. Publicly available raw sequence reads of 720 MDR *K. pneumoniae* isolates were downloaded from the European Nucleotide Archives (ENA) ENA Browser (ebi.ac.uk) (accessed on 20 January 2022). All isolates were of clinical origin, collected between 2011 and 2020, and reported from the six GCCC, namely, Saudi Arabia, *n* = 230; Oman, *n* = 212; Qatar, *n* = 164; UAE, *n* = 98; Kuwait, *n* = 15; and Bahrain, *n* = 1; see Table 1. To ensure high-quality, raw sequencing reads were quality-trimmed and filtered using Trimmomatic (version 0.33) [68]. De novo assembly of reads was performed using SPAdes (ve3.12.0) [69]. The mean number of contigs was 175 (range: 33–2278) for a mean total genome size of 5.6 Mbp (range: 5.0–6.3 Mbp). The mean N50 contig length was 222,214 (range: 5290–729,359) and the mean G+C content was 57% (range: 56–58.2%) (Supplementary Table S1).

In silico antibiotic resistance and virulence gene analysis. The assembled contigs were then annotated, and known antibiotic resistance genes (ARGs) were detected using ResFinder (http://cge.cbs.dtu.dk/services/ResFinder/ (accessed on 15 April 2022)) [70]. The capsular type and O antigen serotype were identified using Pathogen watch (Pathogenwatch | A Global Platform for Genomic Surveillance (accessed on 15 April 2022), the Kaptive online tool (http://kaptive.holtlab.net/ (accessed on 15 April 2022) [71], and Kleborate database (https://github.com/katholt/Kleborate (accessed on 15 April 2022) [72].

Multi-locus sequence typing (MLST). Assembled genomes were typed using both the ‘*Klebsiella pneumoniae*’ database from PubMLST (https://pubmlst.org/abaumannii/ (accessed on 15 May 2022) and Ridom SeqSphere+ v.8.3.5 software (Ridom GmbH, Münster, Germany) [73], and sequence types (STs) were identified using both the Pasteur and Oxford schemes.

Core genome MLST (cgMLST) characterization and phylogenetic analysis. cgMLST analysis was performed using a well-defined scheme available in Ridom SeqSphere+ v.8.3.5 software (Ridom GmbH, Münster, Germany), according to the ‘*K. pneumoniae* sensu lato cgMLST’ version 1.0 scheme (https://www.cgmlst.org/ncs/schema/2187931/ (accessed on 25 September 2022). This included 2358 genes of the *K. pneumoniae* core genome (cgMLST) and 2526 genes of the *K. pneumoniae* accessory genome (wgMLST; total of 4891 targets). Seqsphere+ tool mapped the reads against the reference genome using BWA v 0.6.2 software (parameters setting: minimum coverage of five and Phred value > 30) and defined the cgMLST gene alleles. A combination of all these alleles in each isolate formed an allelic profile that was utilized to create a minimum spanning tree (MST) using Ridom SeqSphere+ with the ‘pairwise ignore missing values; % column difference’ parameter. A threshold was set at ≤15 allelic differences paired with a cluster alert quality threshold of at least 90% good cgMLST targets to define the clusters.

## Figures and Tables

**Table 1:** Characterization of 720 *K. pneumoniae* isolates with the most abundant STs across the six Gulf countries.

Country	Total Number (n)	Carbapenemase Genes							Sequence Types (ST)									
		NDM	KPC	OXA	IMP	VIM	Dual	None	ST 101	ST 11	ST 14	ST 147	ST 15	ST 2096	ST 231	ST 307	ST 45	ST 48
UAE	98	27	6	30	0	0	21	14	0	6	54	10	7	0	9	0	0	1
Saudi	230	21	1	130	0	2	17	59	15	3	43	8	4	98	0	8	6	7
Qatar	164	38	4	27	0	0	10	85	2	5	4	14	2	2	6	8	6	2
Oman	212	77	0	76	0	0	21	38	8	62	3	37	10	0	68	2	0	0
Kuwait	15	2	0	3	0	0	1	9	0	0	0	0	2	0	8	0	0	0
Bahrain	1	0	0	1	0	0	0	0	0	0	1	0	0	0	0	0	0	0
Total	720	165	11	266	0	2	70	206	25	76	105	69	25	100	91	18	12	10
CRKp %	71% (514/720)	32% (165/514)	2% (11/514)	51.7% (266/514)	0	0.38% (2/514)	13.6% (70/514)	28.6% (206/720)	88% (22/25)	94.7% (72/76)	80% (84/105)	84% (58/69)	84% (21/25)	87% (87/100)	73.6% (67/91)	38.8% (7/18)	50% (6/12)	40% (4/10)

**Table 2:** Carbapenem resistance genes identified among the 514 CRKP isolates across the Arabian Peninsula.

Carbapenem Resistance Genes	Total Isolates (n)	Percentage (%)
KPC-2	10	1.94
KPC-2/OXA-232	1	0.19
KPC-3	1	0.19
NDM-1	159	30.93
NDM-1/OXA-162	4	0.77
NDM-1/OXA-181	1	0.19
NDM-1/OXA-232	31	6.03
NDM-1/OXA-48	21	4.08
NDM-5	5	0.97
NDM-5/OXA-181	3	0.58
NDM-5/OXA-232	6	1.16
NDM-5/OXA-48	3	0.58
NDM-7	1	0.19
OXA-162	5	0.97
OXA-181	19	3.69
OXA-232	143	27.82
OXA-48	99	19.26
VIM-29	2	0.38

**Table 3:** Prevalence of capsular type (K locus) and O antigens among major clones of the whole isolate collections.

		ST-14	ST-231	ST-2096	ST-11	ST-147	ST-15	ST-101	ST-45
Capsular type	KL5	1%	--	--	--	--	--	--	--
	KL2	61%	--	--	--	--	4%	--	--
	KL10	--	--	--	--	4%	--	--	--
	KL14	--	--	--	76%	--	--	--	--
	KL15	--	--	--	8%	--	--	--	--
	KL16	1%	--	--	--	--	--	--	--
	KL17	--	--	--	--	--	--	88%	--
	KL19	--	--	--	--	--	24%	--	--
	KL24	--	--	--	12%	--	20%	--	8%
	KL43	--	--	--		--	--	--	50%
	KL47	--	--	--	1%	--	--	--	--
	KL48	--	--	--	--	--	24%	--	--
	KL50	1%	--	2%	--	--	--	--	--
	KL51	--	100%	--	--	--	--	--	--
	KL52	--	--	--	--	--	--	--	8%
	KL64	30%	--	93%	1%	94%	4%	4%	
	KL102	--	--				4%		8%
	KL107	--	--	4%	1%	1%	--	4%	8%
	KL112	--	--	--	--	--	16%	--	--
	KL127	--	--	--	--	--		--	8%
	KL135	1%	--	--	--	--		--	--
	KL166	--	--	1%	--	--	4%	--	--
O antigen	O1	85%	99%	96%	--	--	92%	92%	--
	O2a	10%	1%	3%	13%	91%	4%	4%	67%
	O3	1%	--	--	41%	4%	4%	--	8%
	O4	--	--	--	8%	--	--	--	
	OL101	--	--	--	1%	--	--	--	25%
	OL102	--	--	1%	1%	3%	--	--	--
	OL104	--	--	--	36%	--	--	--	--

## Data Availability

Raw genome sequence data examined in this study are publicly available from the European Nucleotide Archive under the accession numbers stated in Supplementary Table S1. Using the Short Read Archive (SRA) database, metadata on collection date are also available, and the sequencing was done with Illumina platforms (Supplementary Table S1).

## Supplementary Materials

The following supporting information can be downloaded at: https://www.mdpi.com/article/10.3390/antibiotics12071081/s1. Table S1: Genomic characterization, epidemiology, and carbapenemase genes description of 720 Klebsiella pneumoniae collected from six countries around the Arabian Peninsula.
